# Resveratrol: An Antiaging Drug with Potential Therapeutic Applications in Treating Diseases

**DOI:** 10.3390/ph2030194

**Published:** 2009-12-15

**Authors:** Antoni Camins, Felix Junyent, Ester Verdaguer, Carlos Beas-Zarate, Argelia E. Rojas-Mayorquín, Daniel Ortuño-Sahagún, Mercè Pallàs

**Affiliations:** 1 Unitat de Farmacologia i Farmacognòsia i Institut de Biomedicina (IBUB), Centro de Investigación de Biomedicina en Red de Enfermedades Neurodegenerativas (CIBERNED), Facultat de Farmàcia, Universitat de Barcelona, Nucli Universitari de Pedralbes, Av. Joan XXIII s/n 08028 Barcelona, Spain; 2 Departamento de Biología Celular y Molecular, CUCBA, Universidad de Guadalajara, División de Neurociencias, Centro de Investigación Biomédica de Occidente, IMSS, Sierra Mojada 800, Col. Independencia, Guadalajara, Jalisco 44340, Mexico; 3 UMR 975 INSERM, Thérapeutique Expérimentale de la neurodégénérescence, Centre de recherche de l'Institut du cerveau et de la moelle épiniére (CRICM), Université Pierre et Marie Curie, Hôpital de la Salpêtrière, 47 bld de l'Hôpital, 75651 Paris Cedex 13, Paris, France; 4 Laboratorio de Desarrollo y Regeneración Neural, Instituto de Neurobiología, Departamento de Biología Celular y Molecular, C.U.C.B.A, Universidad de Guadalajara, Guadalajara, Jalisco, México

**Keywords:** resveratrol, Alzheimer’s disease, Parkinson’s disease, SIRT1

## Abstract

The prevention of aging is one of the most fascinating areas in biomedicine. The first step in the development of effective drugs for aging prevention is a knowledge of the biochemical pathways responsible for the cellular aging process. In this context it seems clear that free radicals play a key role in the aging process. However, in recent years it has been demonstrated that the families of enzymes called sirtuins, specifically situin 1 (SIRT1), have an anti-aging action. Thus, the natural compound resveratrol is a natural compound that shows a very strong activation of SIRT1 and also shows antioxidant effects. By activating sirtuin 1, resveratrol modulates the activity of numerous proteins, including peroxisome proliferator-activated receptor coactivator-1α (PGC-1 alpha), the FOXO family, Akt (protein kinase B) and NFκβ. In the present review, we suggest that resveratrol may constitute a potential drug for prevention of ageing and for the treatment of several diseases due to its antioxidant properties and sirtuin activation.

## Introduction

One of the hallmarks of pharmacological science is the development of safe drugs for the treatment of diseases. One proposed strategy for the treatment of neurodegenerative disorders that has gained considerable attention is the use of natural antioxidant agents, since one common advantage of all these compounds is their human safety [1]. A considerable amount of research has been conducted in humans with dietary vitamins E and C [2]. In patients with moderately severe Alzheimer’s Disease the administration of vitamin E led to a slight delay of disease progression, thus providing evidence for the beneficial effects of this vitamin as part of a potential treatment in AD [2,3]. In keeping with these results, other studies with vitamin C, carotenoids and other antioxidants in AD patients have argued that these antioxidants might also have a protective effect against this disease [5,6]. Thus, antioxidant drugs could have a potential application in neuropharmacology. In this context, attention has turned to resveratrol (RESV), a naturally-occurring polyphenolic compound with strong antioxidant properties and abundantly found as a component of red wine [7,8,9]. Research has described several beneficial properties of this compound, including anti-carcinogenic, anti-ageing, neuroprotective, analgesic, anti-diabetic and anti-obesity effects [9,10,11,12,13,14,15,16,17,18]. The aim of the present review is to discuss the potential beneficial effects of RESV in the treatment of neurological disorders, not only as concerns its antioxidant properties, but also through the activation of sirtuin 1 [18,19,20,21,22,23,24,25,26].

## Resveratrol as an Antioxidant Drug

One leading theory about the causes of neurodegenerative diseases and aging suggests that free radical damage and oxidative stress play a major role in the pathogenesis of all such diseases, for example, Parkinson’s and Alzheimer’s diseases [27,28,29,30,31]. Oxidative stress is known to induce intracellular cell damage that affects all the biological components such as DNA, lipids, sugars and proteins [1,4,5,27,30,32,33]. Therefore, the imbalance between intracellular ROS and antioxidant defence mechanisms results in oxidative stress that is harmful for neurons. 

RESV (3,5,4’-trihydroxystilbene), the main non-flavonoid polyphenol found in black grapes and red wine, is characterized as a phytoalexin and is produced by a variety of plants in response to stress. It was used as a natural plant compound in traditional Chinese and Japanese medicine) [7,8,9]. Although interest in this compound was initially almost exclusively focused on its antioxidant properties, it has since been reported to possess a wide range of other biological and pharmacological activities including anti-inflammatory, anti-mutagenic, and anti-carcinogenic effects [10,11,12,13,14]. 

Experimental studies indicate that RESV increased the plasma antioxidant capacity and decrease lipid peroxidation in rats [8]. Its strong antioxidant properties have been associated with the beneficial effects of red wine consumption in protecting against coronary heart disease [7,8,14]. Moreover, in spontaneously hypertensive rats RESV significantly reduced markers of oxidative stress such as 8-hydroxyguanosine in urine [7,9,17]. Likewise, in guinea pigs RESV induced catalase activity in cardiac tissue and decreased the concentration of reactive oxygen species (ROS) generated by menadione [14]. These results indicate that resveratrol can suppress pathological increases in the peroxidation of lipids and other macromolecules in vivo, and also that these effects are direct, or the result of upregulating endogenous antioxidant enzymes [8,14].

## Resveratrol and Sirtuin 1 Activation

It has been proposed that the protective mechanism related with RESV neuroprotection, namely as an agonist of the sirtuins (also called SIRT—or silent information regulator two—proteins), which belong to the histone deacetylase family [31,33,34,35,36,37], is independent of its antioxidant properties. Current research is therefore focused on understanding the mechanisms involved in the ability of RESV to increase the activity of sirtuin 1 (SIRT1) and the intracellular pathways activated or regulated by SIRT1. 

Likewise, interest in SIRT1 has also intensified due to its role as a longevity factor in multiple model organisms [35,36]. AS mentioned, SIRT1 belongs to the histone deacetylases (HDACs) family that have been divided into four groups [35,36,37]. Class I and II HDACs are similar to the yeast Rpd3p and Hda1p proteins. Class III HDACs share common features with the yeast transcriptional repressor Sir2p and are referred to as sirtuins [17]. Class I and II HDACs are characterized by their sensitivity to inhibition by trichostatin A (TSA), whereas the characteristic feature of class III HDACs is that they are nicotinamide adenine dinucleotide (NAD^+^)-dependent. A fourth class includes the deacetylase HDAC119 L. Class III histone deacetylases were named after the founding member, the *Saccharomyces* *cerevisiae* silent information regulator 2 (Sir2) proteins [6]. Analyses of SIRT1 enzymatic activity has revealed that it functions differently from previously-described histone deacetylases. Studies using purified SIRT1 revealed that for every acetyl lysine group removed, one molecule of NAD^+^ is cleaved, and nicotinamide and O-acetyl-ADP-ribose are produced [6,7,8]. Therefore, SIRT1 appears to possess two enzymatic activities: The deacetylation of a target protein and the metabolism of NAD^+^. These two activities suggest that SIRT1 could act as a metabolic or oxidative sensor, regulating cellular machinery based on such information [8]. Therefore, it can be hypothesized that the benefits of RESV are due either to its antioxidant properties or to a specific activation of SIRT1, which is involved in responding to molecular damage and metabolic imbalances [17].

## Resveratrol and Aging

As mentioned earlier, in yeast, worms and flies extra copies of the genes that encode sirtuins are associated with extended lifespan [34]. Inbred knockout mice that lack SIRT1 show developmental defects and have a low survival rate and a significantly shorter lifespan compared with wild-type mice, although out breeding seems to improve the phenotype significantly [16,18]. It has been postulated that the main function of sirtuin proteins is to promote survival and stress resistance in times of adversity. An evolutionary advantage arising from the ability to modify lifespan in response to environmental conditions could have allowed these enzymes to be conserved among all species, and to take on new functions in response to new stresses and demands on the organism. This could explain why the same family of enzymes has dramatic effects on lifespan in disparate organisms with seemingly dissimilar causes of aging.

An *in vitro* screen for activators of SIRT1 identified RESV as the most potent of 18 tested inducers of deacetylase activity. Subsequent work has shown that RESV extends the lifespan of *S. cerevisiae*, *Caenorhabditis* *elegans* and *Drosophila melanogaster*, but only if the gene that encodes SIR2 is present in these organisms [16,17]. More recently, RESV was shown to extend the maximum lifespan of a species of short-lived fish by up to 59%, concomitant with the maintenance of learning and motor function with age and a dramatic decrease in aggregated proteins in elderly fish brains; however, the extent to which this effect is Sir2-dependent, if at all, is not known [38,39,40]. The importance of substrate choice *in vitro* highlights the possibility that RESV might alter the substrate specificity of SIRT1 *in vivo*. Indeed, this is the case in *Caenorhabditis* *elegans*, where RESV treatment has been shown to have SIR-2-dependent effects that are substantially different from those obtained by simple over-expression. At all events, the question of whether enhanced SIRT1 activity and/or RESV treatment will increase mammalian lifespan and be of use in ageing-research therapy remains unresolved.

As pointed out above, one important mechanism that controls or favours the process of aging is the dramatic increase in oxidative stress. Previous studies suggest that antioxidant enzymes such as catalase and superoxide dismutase (SOD) could induce lifespan extension. However, the efficacy of antioxidant drugs as anti-ageing molecules is unclear because the lifespan extensions could be abolished in the presence of ROS [8,17]. Thus, it could be argued that the potential beneficial effects of RESV on ageing are not exclusively mediated by its antioxidant properties. 

Recent studies have also demonstrated that the positive effects of RESV on aging tissues, specifically brain, heart and skeletal muscle, were not associated with an increase in SIRT1 expression. Thus, it has been proposed that the anti-aging effects of RESV may be independent of SIRT1 activity. At all events, the complexity of the mechanism of action of RESV is increasing, since recent data suggest that the administration of RESV does not reduce the levels of oxidative stress markers of DNA damage. Therefore, it is also not clear how RESV and dietary restriction increase life span, although it has been proposed that mTOR activation might constitute an additional pathway involved in this process that is activated by food deprivation. Further in-depth studies are required regarding the potential interaction between RESV and mTOR activation [24]. 

### Resveratrol and Neurodegenerative Diseases

Research has described several important roles for SIRT1 in the central nervous system (CNS), mainly in terms of neuronal development and neuroprotection [6,17]. Previous studies demonstrating high SIRT1 levels in the embryonic brain suggest that it might have a role in neuronal and/or brain development. This idea is consistent with some of the phenotypes associated with SIRT1 knockout mice, who show developmental defects and in which postnatal survival is infrequent [34].

As in other mammalian cells SIRT1 promotes survival and stress tolerance in central nervous system (CNS) neurons. In the adult rat brain, SIRT1 can be found in the hippocampus, cerebellum and cerebral cortex. Interestingly, SIRT1 expression is regulated by oxidative stress, since the antioxidant vitamin E has been shown to reduce oxidative damage and reduction of SIRT1 caused by a high fat and sugar diet, with the subsequent restoration of SIRT1 levels [6]. This study suggests that SIRT1 levels in the brain are affected by oxidative stress and energy homeostasis. An interesting recent study employing organotypic hippocampal slice culture as an *in vitro* model of cerebral ischemia showed that RESV given as pre-treatment mimics ischemic preconditioning via SIRT1 [41]. When SIRT1 was inactivated by sirtinol after ischemic preconditioning or RESV pre-treatment, neuroprotection was abolished. Therefore, this study demonstrates a neuroprotective role for SIRT1 in ischemic injury, which could be elicited by a small molecule such as RESV, and is therefore of substantial clinical interest. 

## Resveratrol and Huntington’s Disease

The neurotoxin 3-nitropropionic acid, a mitochondrial complex II inhibitor is a well-established experimental model of Huntington’s disease. Previous studies have reported that the beneficial effects of RESV against this neurotoxin might be attributed to its antioxidant activity [42]. However, several findings in particular suggest that the neuroprotective effects of SIRT1 could be extended to degenerating neurons. Parker *et al*. showed that resveratrol, acting through Sir-2.1 and SIRT1 activation, respectively, protected *Caenorhabditis* *elegans* and mouse neurons against the cytotoxicity of the mutant polyglutamine protein huntingtin. Huntingtin is the product of the gene mutated in the hereditary neurodegenerative disorder Huntington’s disease, where the expansion of a polyglutamine stretch results in a mutant polypeptide that can form cytotoxic aggregates in neurons [43]. Although *Caenorhabditis* *elegans* has no huntingtin orthologue, over-expression of a huntingtin fragment in touch receptor neurons resulted in a gain-of function mechanosensory defect that was able to model the disease. Both RESV and an increased sir-2.1 gene dosage alleviated the worm neuronal dysfunction in a DAF16-dependent manner. Furthermore, RESV decreased cell death associated with neurons cultured from mutant huntingtin (109Q) knock-in mice in a manner that could be reversed by two SIRT1 inhibitors, sirtinol and nicotinamide.

### Resveratrol and Alzheimer’s Disease

A link between SIRT1 and Alzheimer’s disease (AD) is also increasingly evident [6]. The amyloid hypothesis argues that the aetiological agent of AD pathology is extracellular plaques consisting of aggregated beta-amyloid (Ab) peptide generated from proteolytic cleavages of the amyloid precursor protein (APP) [29]. Both intracellular and extracellular soluble oligomeric forms of Ab were shown, in fact, to initiate synaptic malfunctions and the onset of AD symptoms [29,30]. NF-κB signalling in microglia is known to play a critical role in neuronal death induced by Ab peptides [1,5]. The activation of SIRT1 and modulation of NF-κB signalling may result in other beneficial effects such as anti-inflammation, with inflammation being another contributory factor in the neurodegenerative process of this disease.

Likewise, it has been reported that SIRT1 is up-regulated in mouse models of AD. In the inducible p25 transgenic mouse, a model of AD and tauopathies, RESV reduced neurodegeneration in the hippocampus and prevented learning impairment [44]. Furthermore, over expression of SIRT1 via a lentivirus in the hippocampus of p25 transgenic mice conferred significant neuroprotection. Accordingly, an increase in SIRT1 activation may be a potential target for the treatment of neurodegenerative disorders. Another possible link between SIRT1 and AD comes from the potential benefits of CR (RESV mimics the effects of CR) on AD symptoms and progression. It is well known in the epidemiology of neurodegenerative diseases that the incidence of both sporadic Parkinson’s and Alzheimer’s disease is correlated with multiple genetic factors, diet and social behaviour [6,30,31,42]. It has been hypothesised that high calorie diets are associated with an increased risk of AD, and caloric restriction (CR) has been proposed to protect against both PD and AD [35,45]. Firmer evidence for this idea was obtained when Patel *et al*. showed that short-term CR substantially decreased the accumulation of Ab plaques in two AD-prone APP/presenilin transgenic mice lines, and also decreased gliosis marked by astrocytic activation. In another study, it was showed that a CR dietary regimen prevents Ab peptide generation and neuritic plaque deposition in the brain using a mouse model of AD (Tg2576 mice) [46]. The authors suggested that CR resulted in the promotion of APP processing via the non-amyloidogenic α-secretase-mediated pathway. They observed a significant increase in the concentration of brain sAPPα (a product of α-secretase cleavage) and in ADAM10 (a putative α-secretase) levels in CR animals compared to controls. Furthermore, it was demonstrated that CR reduced the content of Ab in the temporal cortex of squirrel monkeys, in a manner that was inversely correlated with SIRT1 protein concentrations in the same brain region [45]. 

The potential link between RESV and AD is also supported by studies which suggest that moderate consumption of wine is associated with a lower incidence of AD and improved neuropathology in a mouse model of the disease [47,48,49,50,51]. Different *in vitro* and *in vivo* studies have investigated the molecular neuroprotective mechanisms associated with RESV. β-amyloid peptide induces cell death through apoptosis in many cell types via reactive oxygen species and such an effect was also blocked by RESV [49,50,52]. More interestingly, RESV can rescue hippocampal primary neurons and PC12 cells from the toxicity of β-amyloid peptide [49]. Thus, a treatment with RESV, administrated 2 h after the amyloid β peptide (25–35) (20 μM), significantly attenuated the amyloid β peptide-induced cell death in a concentration-dependent manner [50]. As regards the other potential pathways involved in RESV neuroprotection against β-amyloid, research has demonstrated the role of PKC activity in this effect. In contrast, studies in SH-SY5Y neuroblastoma cells showed that RESV can induce the activation of the MAP kinases, ERK1 and ERK2 [47]. Recently, in two different APP695-transfected cell lines (HEK293 and N2A), RESV (20–40 μM) could markedly reduce the secretion of the amyloid β peptide (1–40) [52]. This effect of RESV occurred without directly affecting β and γ-secretases, since RESV has no effect on these enzymes [52,53]. The reduction of amyloid β peptide secretion could be due to an increase in its degradation. Metalloendopeptidases such as neprilysin or endothelin-converting enzyme ECE-1 or ECE-2 are candidates for the amyloid β peptide-degrading enzyme in brain [53,54,55], although RESV did not promote the decrease of amyloid β peptide through the activity of these metalloendopeptidases. It has been proposed that the amyloid β peptide itself may lead to proteasome inhibition [55], suggesting that high levels of the amyloid β peptide in the brain of Alzheimer’s patients may inhibit the proteasome and block the degradation of regulators of its own clearance. The treatment of cells with the selective proteasome inhibitors lactacystin, Z-GPFL-CHO or YU101 significantly blocked the RESV-induced decrease of the amyloid β peptide [52]. These results suggest that RESV can activate the proteasome involved in the degradation of the amyloid β peptide. Recently, it has been suggested that RESV is an inhibitor of acetylcholinesterase, and this new pharmacological effect lends support to the potential application of RESV in AD [48].

## Resveratrol and Parkinson’s Disease

Parkinson’s disease (PD) is a neurodegenerative disease characterized at the clinical level by bradykinesia, tremor and rigidity and at the cellular level by a loss of dopamine neurons in grey matter and the frequent presence of intraneuronal inclusions named Lewy bodies, which are mainly composed of α-synuclein [56,57]. Like AD, the familial form of PD concerns only a small proportion of patients (10%). The majorities of them suffer from a sporadic form, and although the genetic causes are fairly well identified the reasons for the emergence of these sporadic forms remains unclear. The involvement of mitochondrial dysfunction in PD has been established for over two decades, since it was discovered that the administration of 1-methyl-4-phenyl-1,2,3,4-tetrahydropyridine (MPTP) causes the emergence of parkinsonism in both laboratory animals and humans through its active metabolite ion MPP+, which inhibits complex I in the chain of mitochondrial electron transfer [57,58]. Complex I inhibition is known to be the major source of free radicals, and it is thought that the alteration in its functions could, above and beyond the declining production of ATP, give rise to increased oxidative stress, thus explaining the emergence of the disease [58]. 

Previous *in vivo* studies suggest that RESV exerts beneficial effects in experimental models of PD [59,60,61]. For example, the administration of a diet containing RESV or treatment with RESV to adult mice prior to treatment with the neurotoxin MPTP exerts neuroprotective effects on dopaminergic neurons [56]. Furthermore, *in vitro* studies have also demonstrated the neuroprotective effects of RESV with different neurotoxins [59,60,61]. However, it has been suggested that SIRT1 activation does not play a major role in the protective effect of RESV against MPP^+^cytotoxicity, because sirtuin inhibitors such as nicotinamide and sirtinol did not counteract neuroprotection by RESV [58]. In contrast, all studies in this area propose that antioxidant actions are responsible for the neuroprotection by RESV against MPP^+^ [58,59]. However, there are recent reports that genetic inhibition of SIRT2 via small interfering RNA rescued α-synuclein toxicity. Furthermore, inhibitors of this enzyme protected against dopaminergic cell death both *in vitro* and in a Drosophila model of Parkinson’s disease, and it has also been shown that inhibition of SIRT2 (another sirtuin protein) rescued α-synuclein toxicity and modified inclusion morphology in a cell model of Parkinson’s disease [60]. At all events, increased SIRT2 expression or activity delays the toxic effects induced by α-synuclein, the protein that forms insoluble aggregates in several age-onset pathologies including Parkinson’s disease. Accordingly, RESV could be an interesting candidate for potential application in the treatment of PD, although probably only on the basis of its antioxidant properties; at present it remains to be clarified if RESV could activates SIRT1 and offers neuroprotection in PD.

## Conclusions

In the last decade, sirtuin biology has come a long way from the original description of yeast NAD+-dependent class III HDACs, which control yeast lifespan. Hence, modulating the expression of SIRT1 or its activity by using sirtuin-activating compounds such as RESV will have pleiotropic effects. SIRT1 is a major modulator of metabolism and also seems to be endowed with neuroprotective activities, as suggested by research with models of PD or AD [62,63,64,65]. Furthermore, other sirtuins might play important roles in some diseases, as illustrated by SIRT2, which could be involved in the treatment of both cancer and PD. Interestingly, low doses of RESV exert the same beneficial effects as caloric restriction in mice [66,67,68,69]. Thus, although dietary restriction is not an appropriate strategy for the treatment of neurological disorders, its beneficial effects could be obtained via RESV [64]. Obviously, further studies in both animal models and humans are needed to define the exact role of sirtuins in the pathophysiology of human diseases. However, it is reasonable to assume that therapeutic interventions that aim to activate or block sirtuins, depending on the context, will one day become useful in the treatment of human diseases.
